# Mutational Spectrum Analysis of Neurodegenerative Diseases and Its Pathogenic Implication

**DOI:** 10.3390/ijms161024295

**Published:** 2015-10-14

**Authors:** Liang Shen, Hong-Fang Ji

**Affiliations:** Shandong Provincial Research Center for Bioinformatic Engineering and Technique, Shandong University of Technology, Zibo 255049, China; E-Mail: shen@sdut.edu.cn

**Keywords:** neurodegenerative diseases, conformation change, mutation, distribution pattern, pathogenesis

## Abstract

One of the most conspicuous features of neurodegenerative diseases (NDs) is the occurrence of dramatic conformation change of individual proteins. We performed a mutational spectrum analysis of disease-causing missense mutations in seven types of NDs at nucleotide and amino acid levels, and compared the results with those of non-NDs. The main findings included: (i) The higher mutation ratio of G:C→T:A transversion to G:C→A:T transition was observed in NDs than in non-NDs, interpreting the excessive guanine-specific oxidative DNA damage in NDs; (ii) glycine and proline had highest mutability in NDs than in non-NDs, which favor the protein conformation change in NDs; (iii) surprisingly low mutation frequency of arginine was observed in NDs. These findings help to understand how mutations may cause NDs.

## 1. Introduction

Protein destabilization is a common mechanism by which amino acid substitutions cause human diseases. Neurodegenerative diseases (NDs), including Alzheimer’s disease, Parkinson’s disease, prion diseases, *etc.*, are a group of chronic disorders characterized by progressive nervous system dysfunction. One of the most conspicuous features of NDs is the occurrence of dramatic conformation change of individual proteins and thus, these diseases are also known as “neurodegenerative conformational diseases”.

Many NDs are reported to be caused by genetic mutations as specific substitutions of one amino acid to another can exert their deleterious effects by compromising protein structure and/or function. At the molecular level, this arises from an inability of the mutant residue to fulfill the roles of the wild-type amino acid. For instance, of the known disease-causing missense mutations released in Human Gene Mutation Database [1], the vast majority (up to 80%) resulted in protein destabilization [2]. As dramatic conformational changes are involved in individual proteins in NDs, it is rational to speculate that the missense mutations associated with these diseases should engender more deleterious changes in protein structural stability than those in other non-conformational diseases. Thus, elucidating the underlying patterns of the disease-causing missense mutations in NDs can provide useful clues for understanding their pathogenesis. Here, we conducted a systematic analysis of a mutational spectrum of disease-causing missense mutations in NDs at both the nucleotide and amino acid levels. Further, we also performed a parallel analysis of the missense mutations annotated as pathogenic in the Single Nucleotide Polymorphism database (dbSNP) to represent the status in other human diseases (non-NDs). Through comparing the two data sets, we found some distinct differences in both the type and frequency of missense mutations between NDs and non-NDs.

## 2. Results and Discussion

### 2.1. Distribution Patterns of Pucleotide Substitutions

**Figure 1:**
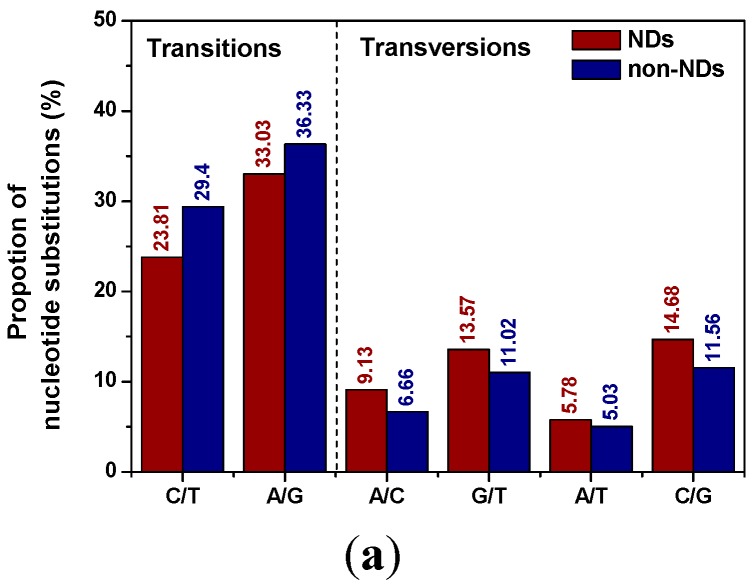

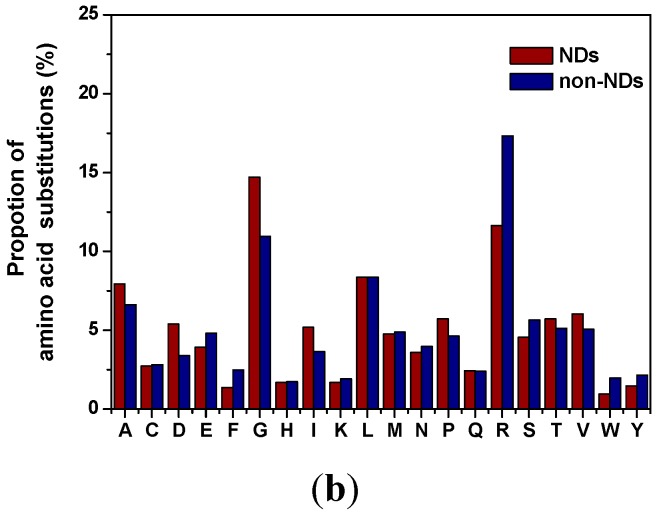
(**a**) The distribution of nucleotide substitution types and their proportions of NDs in comparison with non-NDs; (**b**) The amino-acid substitution spectrum of disease-causing missense mutations in NDs and non-NDs.

The distribution of nucleotide substitution types and their proportions of NDs in comparison with non-NDs were illustrated in Figure 1a. It was found that the distribution patterns of nucleotide substitutions were uneven. The transition substitutions A/G and C/T (A/G represents the sum of all A→G and G→A substitutions and so on) in NDs (56.84%) was lower compared to that in non-NDs (65.73%). Further, the transition substitutions A/G and C/T both decreased, while all the transversion substitutions, *i.e.*, A/C, G/T, A/T, C/G, increased. The ratio of transitions over transversions (Ts/Tv) showed that NDs displayed a lower overall ratio than non-NDs (1.31 for NDs *vs.* 1.92 for non-NDs), indicating that the differences of transition bias between NDs and other diseases were noteworthy. Transitions are generally favored over transversions among spontaneous mutations, and transversions are associated with functionally important amino acid alterations. Thus, the high proportion of transversion mutations would exert deleterious effects on protein structure and function in NDs in comparison with non-NDs.

The relative frequencies of nucleotide substitutions from A, T, C and G to any other nucleotide were also listed in Table 1. Among the four bases, guanine was the most mutable nucleotide, with a relative frequency of 40.15% in NDs, while A and T were the least mutable ones, with relative frequencies of 18.69% and 15.9%, respectively. Although guanine was also the most mutable nucleotide (40.87%) in non-NDs, its substitution frequencies to A, T, C were markedly different with NDs. For instance, the transition substitution frequency of G→A 27.14% in non-NDs is higher than 22.91% in NDs, while the transversion substitution frequencies of G→T (6.94% *vs.* 8.79%) and G→C (6.79% *vs.* 8.45%) in non-NDs were both lower than those in NDs, respectively. Notably, guanine in genomic DNA is highly susceptible to oxidative stress due to its lowest oxidation potential resulting in the high occurrence of G:C→T:A transversion substitutions [3]. Thus, the ratio of G:C→T:A transversion to G:C→A:T transition mutation can be used to assess the degree of oxidative DNA damage. The ratio from the present study was 0.34 and 0.23 for NDs and non-NDs, respectively, suggesting that the oxidative DNA damage level was much higher in NDs than in non-NDs. Considering significant elevations of the chief guanine oxidation product 8-oxo-7,8-dihydro-guanine in patients brains with NDs, e.g., Alzheimer’s disease, Parkinson’s disease, Huntington’s disease, *etc.*, compared with control groups [4,5,6,7,8,9,10], it can be speculated that excessive guanine-specific oxidative DNA damage may be a potential risk factor for NDs.

**Table 1:** Pattern of nucleotide substitution in neurodegenerative diseases (NDs) and non-NDs ^a^.

From	To				
	A	T	C	G	Total
A	―	3.56 (2.09)	5.01 (2.76)	10.12 (9.19)	18.69 (14.04)
T	2.22 (2.94)	―	8.90 (10.35)	4.78 (4.08)	15.90 (17.37)
C	4.12 (3.90)	14.91 (19.05)	―	6.23 (4.77)	25.26 (27.72)
G	22.91 (27.14)	8.79 (6.94)	8.45 (6.79)	―	40.15 (40.87)
Total	29.25 (33.98)	27.26 (28.06)	22.36 (19.91)	21.13 (18.04)	―

^a^ Table entries are the inferred percentage of nucleotide changes in NDs. Values in parentheses correspond to non-NDs.

### 2.2. Position Distribution Patterns of Nucleotide Substitution

Nucleotide substitutions can occur in three codon positions for each single point mutation; however, changes in the first or second nucleotide position are more likely to alter the encoded amino acid than in the third position. Thus, it is intriguing to explore the frequency of nucleotide substitutions in three codon positions in NDs and non-NDs. As listed in Table 2, the mutation frequency of nucleotides in three codon positions was distributed unevenly. Mutation frequency in second codon position was the highest both in NDs and non-NDs (49.28% *vs.* 52.78%). However, in comparison with non-NDs, the proportion of transition mutation in the second codon position decreased by 6.42% and transversion increased by 2.92% in NDs. In general, codons in the second position with a pyrimidine tend to code for hydrophobic amino acids, while codons with purines usually code for polar amino acids. The transition mutations in the second position will simply replace one amino acid with a chemically similar one, while transversion mutations will alter the chemical property of amino acid. Therefore, based on the results of the present analysis, the increased transversion mutations in the second position in NDs will engender changes of residual chemical property, which may subsequently attenuate the protein structural stability.

**Table 2:** Position distribution patterns of nucleotide substitution in NDs and non-NDs.

Position	NDs			Non-NDs		
	Ts (%)	Tv (%)	Total (%)	Ts (%)	Tv (%)	Total (%)
First	26.97	16.75	43.72	29.79	12.24	42.03
Second	29.04	20.24	49.28	35.46	17.32	52.78
Third	0.67	6.34	7.01	0.47	4.72	5.19
Total	56.68	43.32	100	65.72	34.28	100

### 2.3. Distribution of Amino Acid Substitutions

The amino-acid substitution spectrum of disease-causing missense mutations in NDs and non-NDs were illustrated in Figure 1b. It can be seen that the amino-acid mutation frequencies varied considerably from one another. Among of them, glycine (G) and arginine (R) were the most frequent mutations both in NDs and non-NDs, which was 14.72% and 11.65% in NDs, 10.95% and 17.33% in non-NDs, respectively. Several studies have also found larger contributions of G and R than other amino acids in human genetic diseases [11,12]. Also, consistent with the present results of R and G in non-NDs, mutations at R residue occurred more frequently than mutations at G residue [11,12]. In contrast, in NDs, mutations at G residue had higher mutability than R residues (14.72% *vs.* 10.95%). Then, it was intriguing to explore why R residue exerted low mutability and G residue exerted high mutability in NDs than in non-NDs. Arginine is represented by six codons: CGA, CGG, CGU, CGC, AGG and AGA. Among these, four codons have a CpG dinucleotide that can spontaneously mutate by deamination either to TG or CA dinucleotides, resulting in the very high mutability of arginine [13]. However, Khan *et al.* found that R had clearly the highest relative mutability among the original disease-associated residues, while its mutation frequency within different structure types was distinct [12]. They found that R had significantly higher mutation frequency in the outside secondary structural elements than in α-helices, β-structures, turns and bends [12]. Considering the crucial pathogenic role of protein conformational conversion from α-helix to β-sheet in NDs, we speculated that the mutation of R residue outside secondary structural elements may not cause disease in most cases, which may account for the surprisingly lower disease-causing mutation frequency of R in NDs.

Glycine is a conformational residue and sometimes known as “helix breaker”. Glycine is the only non-chiral amino acid because it contains a very small volume of hydrogen atom as its side chain, which gives G an extraordinary role in making the local peptide structure flexible and adopts a much larger range of conformations than other residues. Thus, the outcome for G mutation will increase the volume of the protein and disturb the protein structural stability. Besides G, proline (P) is another commonly known residue to disrupt secondary structure because in α-helice and β-strand, the introduction of a pyrrolidine ring often causes steric clashes to neighboring residue side chains. As shown in Figure 1b, the mutation of G and P both occurred more frequently in NDs than in non-NDs. Thus, the higher mutation proportion of G and P in NDs than in non-NDs also favored the dramatic conformation changes of NDs.

## 3. Experimental Section

Thanks to the previous endeavor, several comprehensive mutation databases of NDs have been constructed [14,15,16,17,18]. Our interest focused on the mutation data set containing information on single nucleotide polymorphism and a total of seven types of NDs, *i.e.*, Alzheimer’s disease, Frontotemporal dementia, Parkinson’s disease, Wilson’s disease, Amyotrophic lateral sclerosis, Huntington’s disease and prion diseases, were selected in the present analysis. The required mutation data were obtained from five databases including the Alzheimer Disease and Frontotemporal Dementia Mutation Database (AD & FTDMDB) [14], Parkinson’s Disease Mutation Database (PDmutDB) [15], Wilson Disease Mutation Database [16,17], Amyotrophic lateral sclerosis mutation database [18], and two websites regarding prion diseases [19] and Huntington’s disease [20].

In total, 899 non-redundant nonsynonymous single-nucleotide polymorphisms (nsSNPs) associated with NDs were considered. To discriminate the spectrum of disease-causing mutations in NDs *vs.* non-NDs, 5826 non-redundant mutations published in the literature with annotation as pathogenic in the dbSNP until August 2014, were selected as disease-causing missense mutations in non-NDs.

## 4. Conclusion

In summary, a mutational spectrum analysis of disease-causing missense mutations was performed at nucleotide and amino acid levels and compared with non-NDs. The findings deepened our knowledge on how mutations may cause NDs, including the excessive DNA oxidative damage and dramatic protein conformational changes in NDs.
